# Sunlight-powered photodynamic therapy for painless diabetic wound care

**DOI:** 10.1093/nsr/nwag033

**Published:** 2026-01-16

**Authors:** Yuan Lu, Ke-Bin Huang, Doron Shabat

**Affiliations:** Key Laboratory for Chemistry and Molecular Engineering of Medicinal Resources (Ministry of Education of China), Guangxi Key Laboratory of Chemistry and Molecular Engineering of Medicinal Resources, School of Chemistry and Pharmaceutical Sciences, Guangxi Normal University, China; Key Laboratory for Chemistry and Molecular Engineering of Medicinal Resources (Ministry of Education of China), Guangxi Key Laboratory of Chemistry and Molecular Engineering of Medicinal Resources, School of Chemistry and Pharmaceutical Sciences, Guangxi Normal University, China; School of Chemistry, Raymond and Beverly Sackler Faculty of Exact Sciences, Tel-Aviv University, Israel

Chronic infected wounds are among the most debilitating complications of diabetes, often leading to prolonged hospitalization, antibiotic resistance, severe pain, and even limb amputation [1]. Photodynamic therapy (PDT) offers an attractive non-antibiotic strategy by generating reactive oxygen species (ROS) to eradicate pathogens, yet its clinical translation has been limited by shallow light penetration, treatment-associated pain, phototoxicity, and reliance on expensive laser equipment [2–4]. These limitations have largely confined PDT to device-dependent, clinic-based settings. In a recent study, Gong, Gu, Jiang, Kim and co-workers report a sunlight-activated nanospray that not only improves PDT performance but also suggests a more accessible mode of clinical deployment, offering a painless, portable, and multifunctional therapy for diabetic wound infections [5].

**Figure 1.**
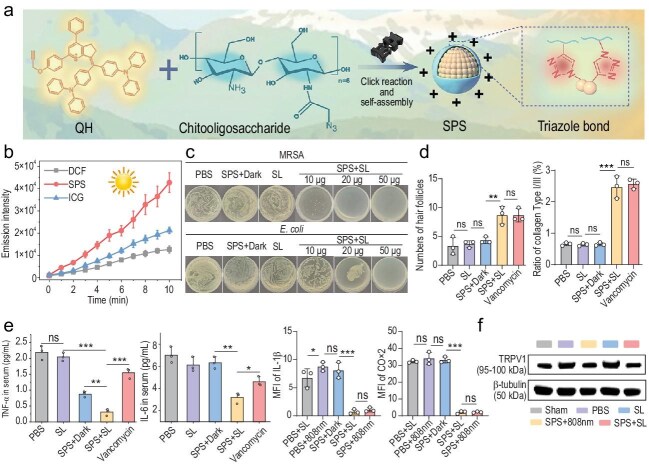
(a) Schematic illustration depicting the fabrication and structural design of the SPS nanospray. (b) Time-dependent variation in DCFH fluorescence intensity in systems containing SPS and ICG under continuous sunlight exposure. (c) Representative images of bacterial colony formation on MRSA- and *E. coli*-inoculated agar plates following different treatments. (d) Quantitative assessment of hair follicle density and collagen I/III ratio in regenerated skin tissues across the indicated groups. (e) Comparative quantification of inflammatory mediators, including TNF-α, IL-6, IL-1β, and COX2, in various treatment groups. (f) Western blot analysis of TRPV1 protein levels in the lumbosacral region of the spinal cord. Reproduced with permission from Ref. [5].

The authors introduce a chitosan oligosaccharide–coated nanoparticle system (SPS) built around a newly designed second near-infrared (NIR-II) photosensitizer (Fig. 1a). Through click chemistry–mediated self-assembly, the photosensitizer is converted into water-dispersible nanoparticles that can be applied directly to wounds as a topical spray. Unlike conventional PDT agents that require controlled laser illumination, SPS is efficiently activated by natural sunlight, producing abundant ROS even under low-intensity ambient light (Fig. 1b). This sunlight-driven activation reduces reliance on specialized instrumentation, broadening the potential contexts in which PDT may be applied.

This reliance on sunlight is more than a technical convenience. By avoiding high-intensity laser irradiation, SPS-mediated PDT substantially reduces pain and thermal damage at the treatment site, a key barrier to patient compliance. Meanwhile, the photosensitizer exhibits favorable photophysical behavior in the NIR-II window, which allows effective light penetration into deeper tissues and promotes efficient type I ROS production. As a result, robust antibacterial efficacy is achieved against both *Staphylococcus aureus* (including MRSA) and *Escherichia coli* (Fig. 1c).

Beyond its antibacterial performance, SPS displays pronounced multifunctional characteristics. The chitosan oligosaccharide coating confers inherent hemostatic capability to the nanospray, enabling rapid bleeding control *in vivo*. In diabetic wound models, SPS administration leads to faster wound closure, improved collagen reorganization, and increased hair follicle regeneration (Fig. 1d), collectively reflecting a shift toward scar-attenuated healing. Consistent with these regenerative outcomes, key inflammatory mediators, including IL-1β, IL-6, TNF-α, and COX-2, are significantly reduced following treatment (Fig. 1e), suggesting that sunlight-triggered PDT can effectively reshape the chronic inflammatory milieu typical of diabetic wounds.

Notably, an unexpected yet clinically relevant analgesic effect is also observed. A combination of behavioral assessments, electrophysiological measurements, and molecular analyses reveals that SPS treatment suppresses TRPV1 expression while restoring spinal neuronal excitability to near-physiological levels (Fig. 1f), thereby alleviating wound-associated pain. This finding highlights an additional therapeutic dimension of PDT beyond antimicrobial action.

By unifying antibacterial, anti-inflammatory, hemostatic, regenerative, and analgesic functions within a single sunlight-responsive nanospray, this study challenges the traditional view of PDT as a technology constrained by complex instrumentation and clinical settings. At the same time, further studies will be required to address practical considerations such as variability in natural light exposure, standardization of dosing, and long-term safety associated with repeated photodynamic activation. Nevertheless, the consistent therapeutic performance of SPS across diverse illumination conditions highlights its promise for emergency applications and use in resource-limited environments.

Taken together, these findings illustrate how rational nanomaterial engineering can extend the practical scope of PDT as a patient-friendly modality. The sunlight-driven SPS nanospray provides a compelling example of next-generation wound care systems that move beyond antibiotic dependence toward multifunctional, non-invasive therapeutic solutions.
